# Crystallisation and characterisation of muscle proteins: a mini-review

**DOI:** 10.1007/s10974-023-09648-2

**Published:** 2023-05-03

**Authors:** Lata Govada, Naomi E. Chayen

**Affiliations:** Division of Systems Medicine, Department of Metabolism, Digestion and Reproduction, Faculty of Medicine, Imperial College London, W12 0NN London, UK

**Keywords:** Crystallisation, X-ray crystallography, Muscle proteins, α-actinin, Myosin-binding protein-C

## Abstract

The techniques of X-ray protein crystallography, NMR and high-resolution cryo-electron microscopy have all been used to determine the high-resolution structure of proteins. The most-commonly used method, however, remains X-ray crystallography but it does rely heavily on the production of suitable crystals. Indeed, the production of diffraction quality crystals remains the rate-limiting step for most protein systems. This mini-review highlights the crystallisation trials that used existing and newly developed crystallisation methods on two muscle protein targets - the actin binding domain (ABD) of α-actinin and the C0-C1 domain of human cardiac myosin binding protein C (cMyBP-C). Furthermore, using heterogenous nucleating agents the crystallisation of the C1 domain of cMyBP-C was successfully achieved in house along with preliminary actin binding studies using electron microscopy and co-sedimentation assays .

## Introduction

It has long been recognised that the function of a protein is directly linked to its three-dimensional structure. Before that can be experimentally determined, however, it is necessary to know the amino acid sequence of that protein. Towards that end the determination of the complete DNA sequences of several genomes, including that of human, has allowed the primary structures of thousands of proteins to be characterised (Chayen 2005). Modern structural biology then has the capability of employing three experimental methods to determine the medium and high-resolution structure of the biological macromolecule concerned. These methods are X-ray crystallography, Nuclear Magnetic Resonance Spectroscopy (NMR) and Electron Microscopy (EM). It is important to note that while the structural information provided by these three techniques is different it is highly complementary, and this has often proved to be invaluable in structural/functional studies. Indeed, the complexity of biology is such that the application of a single technique is often insufficient to yield the answers sought to the level of detail required, whereas integrated approaches have become increasingly commonplace and the results have become correspondingly more detailed (Cerofolini et al. 2019).

EM has proved a versatile tool for determining the overall envelope shape and quaternary structure of proteins and protein complexes. With the recent advances in biological structural EM, protein structures can now be obtained by cryo-EM and single-particle analysis at resolutions that used to be achievable only by crystallographic or NMR methods (Morris and da Fonseca 2017).

Tertiary structure determination of proteins by NMR has proved to be a powerful application of this type of spectroscopy and is one that has allowed characterisation of dynamic processes such as protein folding/unfolding, chemical exchange of target molecules and catalysis. A number of software and web-based resources for NMR data analysis have been developed recently and this has contributed to the systematic integration of sophisticated semi-automated NMR platforms for the structure determination of biomolecules (Sugiki et al. 2017).

X-ray crystallography, however, is currently the most effective and widely used technique for determining the three-dimensional structures of proteins and other macromolecules at high resolutions. It does, nonetheless, require that the protein be present in its most highly ordered form i.e. that of a crystal, (Blow 2002). This then allows, structural studies using X-ray diffraction techniques, often at atomic resolution. Unfortunately, the formation of protein crystals, which involves the purified protein undergoing slow precipitation from an aqueous solution, has remained the major challenge for crystallographers and the success (or otherwise) of crystallisation has remained somewhat of an art. Indeed, the act of crystallisation has generally proved to be the rate-limiting step in structural studies.

Biocrystallisation follows the same rules of crystallisation as inorganic or organic small molecules, but is multi-parametric, making it a far more complex process. The fact that proteins are extremely sensitive to external conditions accentuates this complexity. The three classical steps include nucleation, growth and cessation of growth. Nucleation is, of course, the vital first step upon which crystal growth can only follow. The principle of inducing protein crystallisation depends on the basic strategy of bringing a system into a state of limited supersaturation, this being defined as the ratio of the protein concentration to its solubility value. Super saturation provides the driving force for crystal growth and occurs under non-equilibrium conditions (Ducruix, 1999).

**Fig. 1:**
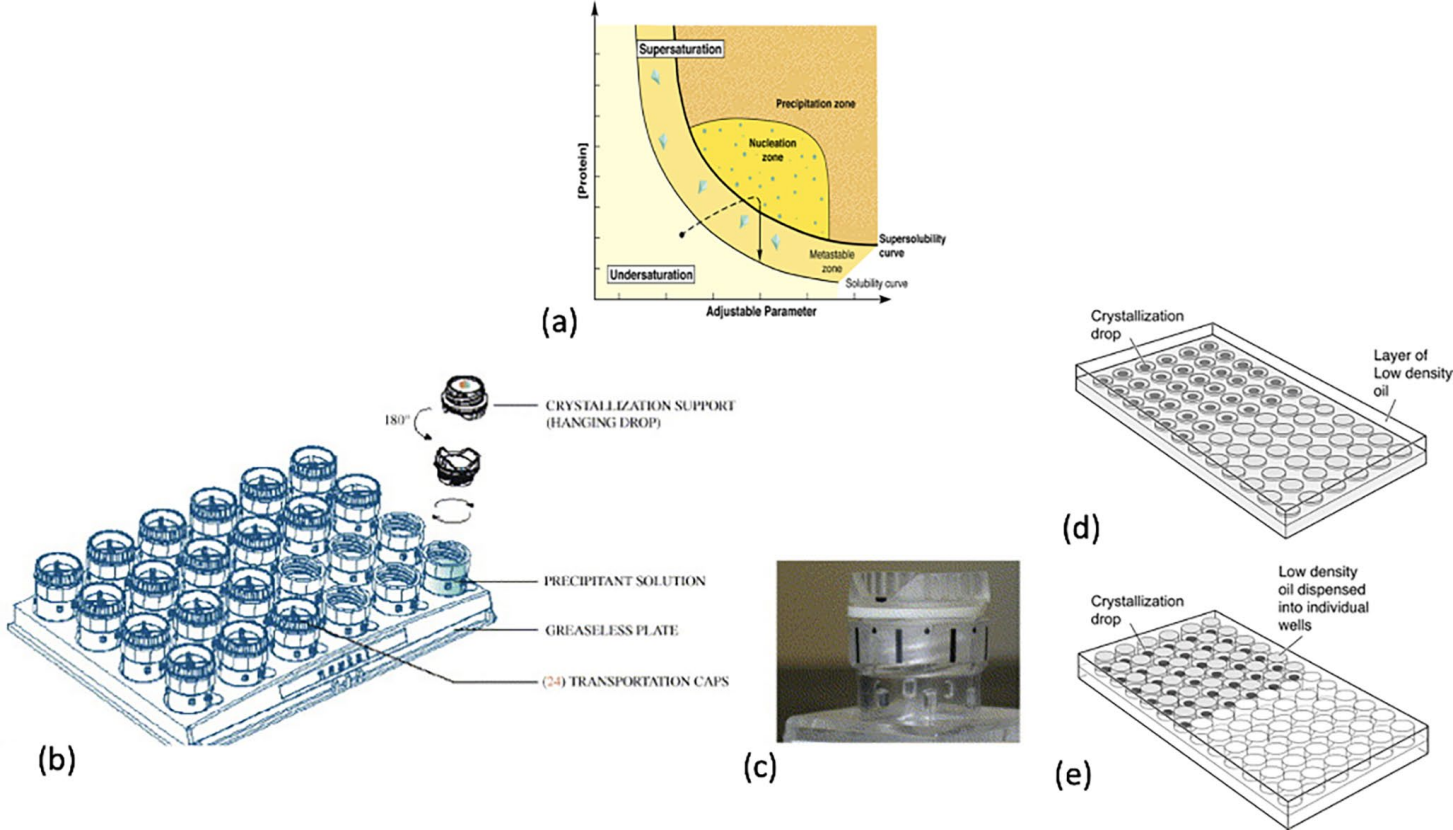
Crystallisation phase diagram and set ups using the modified hanging drop and microbatch methods (**a**) Schematic illustration of a protein crystallisation phase diagram showing that the adjustable parameters are precipitant or additive concentration, pH and temperature (Chayen 2004) (**b**) 24-well crystallisation tool with screwcaps incorporating the coverslips where crystallisation drops are set up, (**c**) the close-up of a single screw cap on its well, (**d**) standard microbatch under oil experiments, (**e**) inverted experiments (Chayen 2005; Khurshid and Chayen 2006)

Phase diagrams form the basis for the design of crystal growth (Fig. 1a). The experimental determination of a phase diagram for a protein under given physical conditions is highly useful and provides a rational approach for choosing the optimum conditions for crystal growth (Saridakis and Chayen 2003). The major concentration areas in the phase diagram are the undersaturation and supersaturation zones. The former represents a theromodynamically stable system that does not favour the crystallisation of biological molecules. In contrast, in the supersaturated zone the macromolecular solution is thermodynamically unstable and has a concentration that is higher than that at equililibrium. Depending on the kinetics used to attain equilibrium, the levels of supersaturation can be further subdivided into three zones:

1. Precipitation zone: Precipitation will occur at high levels of supersaturation.
2. Nucleation zone: This is the region where crystal nuclei first appear. Nucleation is the process by which molecules that are free in solution aggregate in a regular manner to produce thermodynamically stable assemblies with a repeating lattice.
3. Metastable zone: This area of the supersaturation zone does not give rise to nucleation, as the level of critical supersaturation is not attained. Crystals can grow here, however, if nuclei are present, but no new nuclei will be formed (McPherson 1999).

The nucleation event in protein crystallisation is the part of the process that is generally the most poorly controlled. It is widely accepted that the protein should be in the metastable phase for crystal growth, but for nucleation to occur higher levels of supersaturation are needed. Any environment that favours the latter provides a potential nucleation site and may lower the energy barrier for nucleation (Chayen et al. 2001) In many crystallisation experiments, however, sufficiently high levels of supersaturation are not reached to allow the critical nucleation event to occur. However, if an environment can be created that favours a higher local concentration of macromolecules, the energy barrier for nucleation may be lowered. Under such conditions, the introduction of nucleation-inducing agents at lower levels of supersaturation levels may facilitate nucleation and initiate crystal growth. Indeed, inorganic materials, like apophyllite, were amongst the first to be successfully employed as heterogenous nucleants to facilitate nucleation and subsequent crystal growth by epitaxy (Mcpherson and Shlichta 1988). The Chayen group, which was an integral part of the Biological Structure and Function, headed by Prof John Squire at Imperial College London, specialised in the methodology of protein crystallisation (Fig. 1b-e).

Microbatch under oil, a miniaturisation of the batch method and use of mesoporous nucleants, was pioneered by the Chayen group. Porous silicon was the first such nucleant tested successfully (Chayen et al. 2001).

A bio-glass material (CaO-P_2_O_5_-SiO_2_), a highly porous surface with cavities of similar sizes to proteins was developed and this has facilitated the crystallisation of 14 proteins. This is the highest number reported for any single nucleant (Chayen et al. 2006; Saridakis and Chayen 2009) and is commercially available as Naomi’s Nucleant (Molecular Dimensions Ltd -MD2-07). Thereafter, carbon nanotubes (Asanithi et al. 2009) and porous gold foil (Kertis et al. 2010) were also tested for their nucleating properties on different proteins. Bio-glass, carbon nanotubes and porous gold foil were also tested on cMyBP-C. The rationale behind these mesoporous materials is that are likely to constrain protein molecules and thereby encourage them to aggregate in a crystalline order. Although other nucleants such as Molecularly Imprinted Polymers (MIPs) (Khurshid et al. 2015; Saridakis et al. 2011) and, more recently, functionalised carbon nano-materials have been successfully designed and developed for protein crystallisation (Govada et al. 2016, 2022; Leese et al. 2016), these have not been tested thus far on any muscle protein targets.

## Background of muscle architecture

Muscle tissue is a key component in many physiologically important organs in both vertebrates and invertebrates. It has evolved to meet a wide variety of functions and is composed of myofibrils, along which there are the repeating units called sarcomeres. In the vertebrates the muscles may be classified as either smooth or striated, with the latter further subdivided into skeletal and cardiac. Vertebrate cardiac muscle and the various skeletal muscle isoforms are characterised by different mechanical properties designed to best suit their particular physiological demands (Luther et al. 2003). Each sarcomere of the muscle filament is comprised of the myosin-containing thick filaments and the actin-containing thin filaments. Regulatory proteins, such as troponin and tropomyosin, are associated with the actin filaments. In turn, the M-line and C-proteins are associated with the myosin filaments. There are also a number of cytoskeletal proteins, such as titin, nebulin and α-actinin, which help to provide a scaffold for sarcomere building. Structural biology techniques, particularly protein crystallography, electron microscopy and X-ray diffraction, have been used to study the various components of the thick and thin filaments. In contrast, smooth muscle is much less well organised than the striated muscle fibre and is composed of spindle-shaped cells arranged in bundles or sheets.

**Fig. 2:**
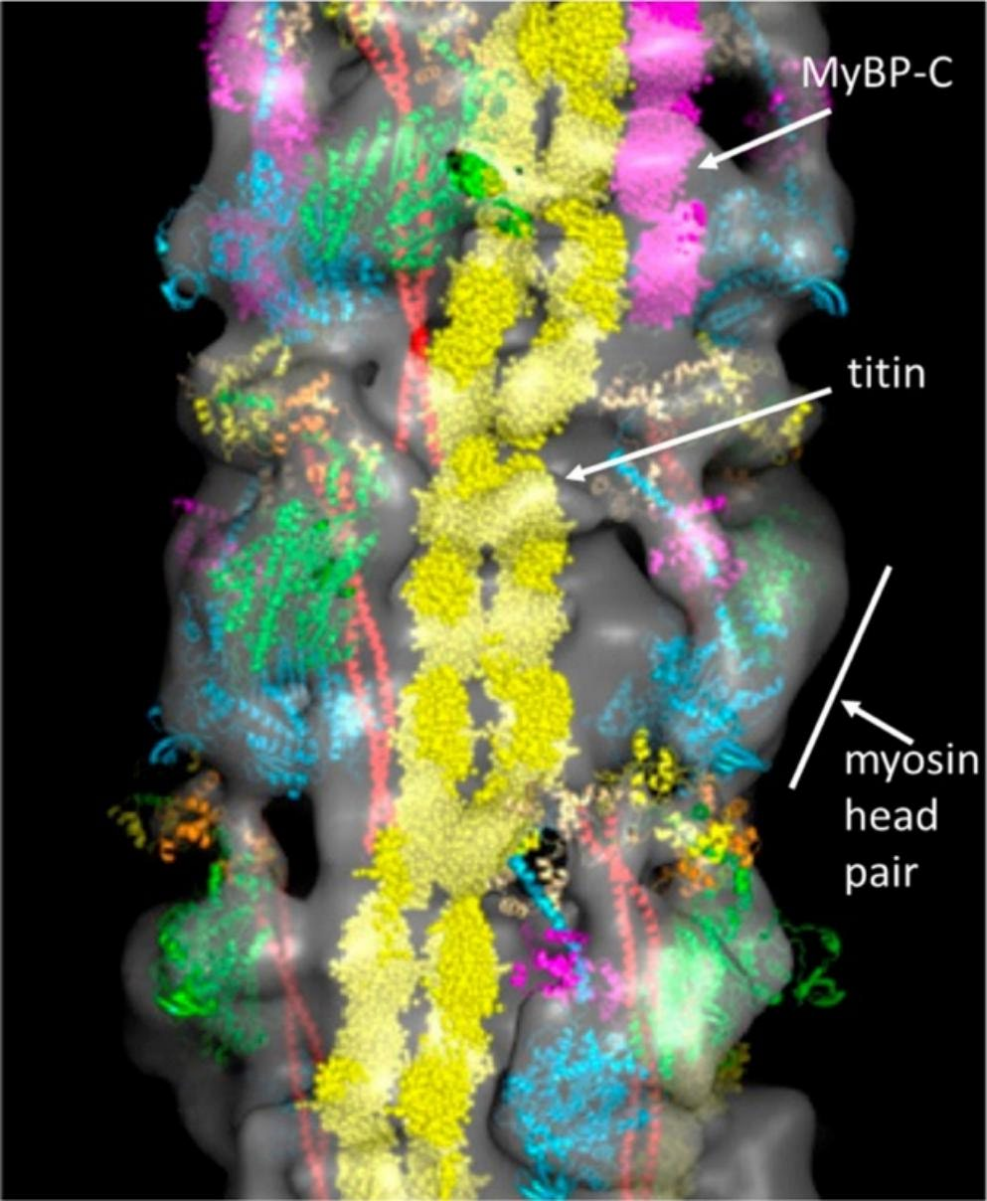
Three-dimensional reconstruction of part of the bridge region of myosin filaments from human cardiac muscle. The possible location of strands of titin and the accessory C protein (MyBP-C) are marked in yellow and mauve respectively (Squire 2019)

Crystallisation trials of two muscle proteins carried out under the guidance of John Squire are reported here:

1. Actin-Binding Domain (ABD) of the chicken smooth muscle α-actinin : α-actinin is an antiparallel homodimer that binds F-actin via the N-terminus of each monomer (Luther and Squire 2002) and is required for the organisation and function of the contractile machinery of muscle. The skeletal, cardiac, and smooth muscle isoforms are localised in the Z-disk and analogous dense bodies (Sjoblom et al. 2008).
2. C0-C1 domain of the human cardiac Myosin-Binding Protein C (cMyBP-C), a component of the myosin filaments of skeletal and cardiac muscles (Fig. 2). This comprises a string of globular domains which includes eight immunoglobulin-like and three fibronectin-like domains termed C0–C10. It binds to myosin and titin, and probably to actin, and may have both a structural and a regulatory role in muscle function. It is also thought to play a role both in the regulation of contractility and in the maintenance of myosin filament structure (Govada et al. 2008).

## Review of crystallisation trials on two muscle proteins

1. ABD of α-actinin

**Fig. 3:**
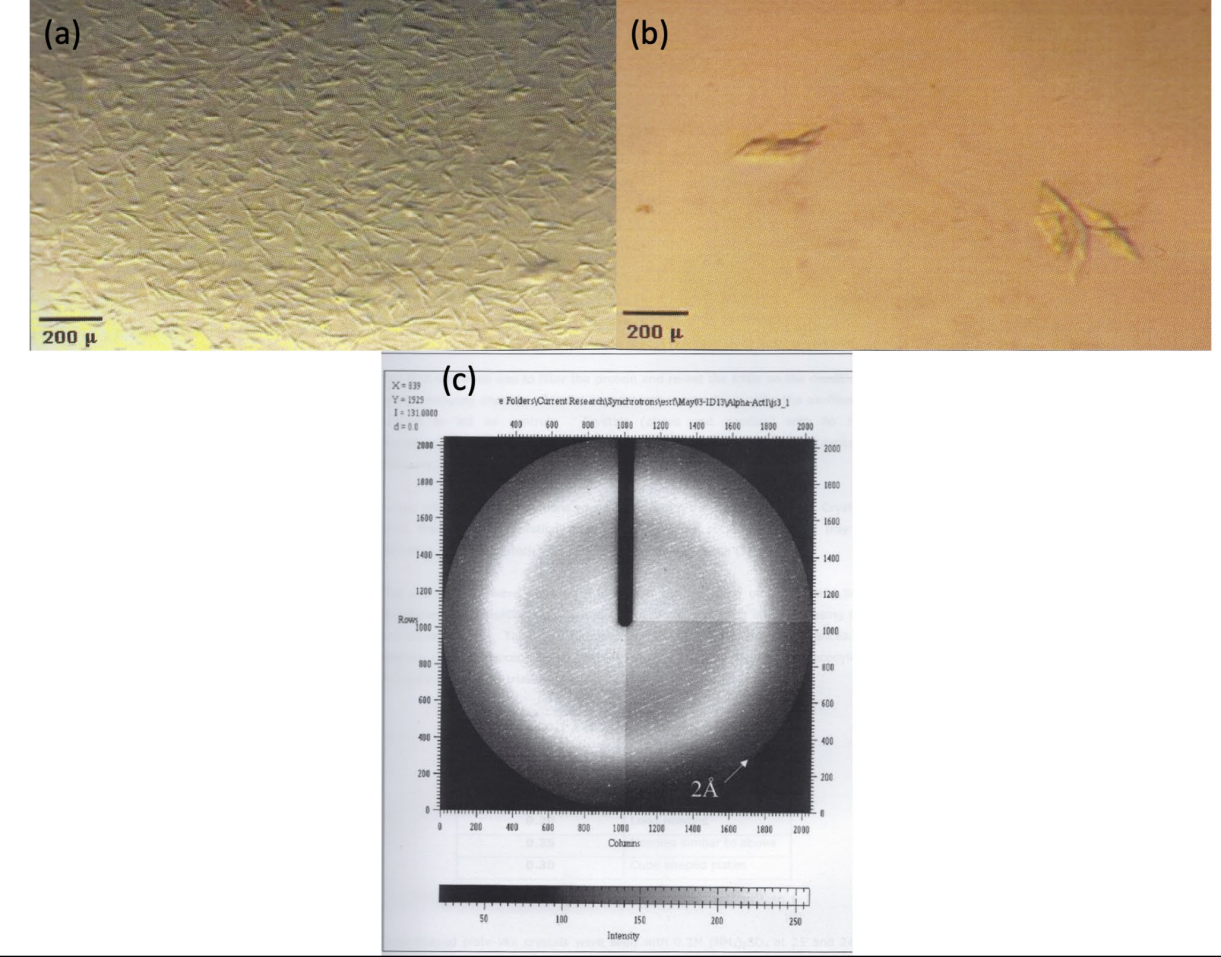
Crystallisation and diffraction analysis of ABD of α-actinin (**a**) initial leads as needles from screening experiments; (**b**) two-dimensional stacks of plates after optimisation; (**c**) diffraction pattern of plates of the ABD of α-actinin (1.9 Å resolution)

About three weeks after setting up the initial screening trials of α-actinin tiny needles were obtained with dimensions of about 100 μm X 20 μm X 5 μm (Fig. 3a). Further optimization resulted in plates stacked together with dimensions of about 200 μm X 50 μm X 5 μm (Fig. 3b). These too were produced about three weeks after setting up the experiments. The crystals were irradiated by X-rays at the home source and produced data to a resolution of 5.4 Å. Although these crystals of α-actinin subsequently diffracted to a higher resolution (1.9 Å) on the ID-13 microfocus beam at ESRF, Grenoble, they exhibited considerable mosaic spread (Fig. 3c). The crystals grew well in two dimensions but failed to grow in the third dimension beyond 5 μm. This precluded a complete dataset being collected. However, the molecular structure of the ABD of human skeletal muscle α-actinin (isoform 3) in two crystal forms was determined (Franzot et al. 2005).

2. Cardiac myosin-binding protein C (cMyBP-C

Initial screening trials of cMyBP-C produced haystacks. Conventional optimsation experiments of these leads produced clustered rods after three weeks. These clustered rods diffracted up to a resolution of about 3 Å. Additional optimisation using the modified hanging drop vapour diffusion trials produced crystals of the C1 domain (Govada and Chayen 2009), which was ultimately solved to a resolution of 1.55Å by Multiple Anamolous Dispersion (MAD) analysis using its seleno-methoinne derivative, with the same conditions (Govada et al. 2008).

Another modified method developed in the Chayen lab, the inverted microbatch experiments produced crystals of the C1 domain which diffracted to 1.3 Å (Fisher et al. 2008).

### Nucleant trials

Crystals of the C1 domain of cMyBP-C were only obtained at metastable conditions in the presence of the Bioglass, porous gold foil and carbon nanotubes (TX-100 bucky paper). Crystals grown on TX-100 buckypaper diffracted to a resolution of 1.6 Å (Asanithi et al. 2009).

### Actin-binding experiments with cMyBP-C

In 2003, Squire et al. were first to show a possible actin-binding sequence in the Pro-Ala-rich N-terminal domain next to C1 in skeletal muscle MyBP- C or between C1 and C0 in cardiac muscle MyBP-C(Squire et al. 2003). Initial co-sedimentation assays and electron microscopy experiments with F-actin were subsequently carried out and corroborated the above finding.

**Fig. 4:**
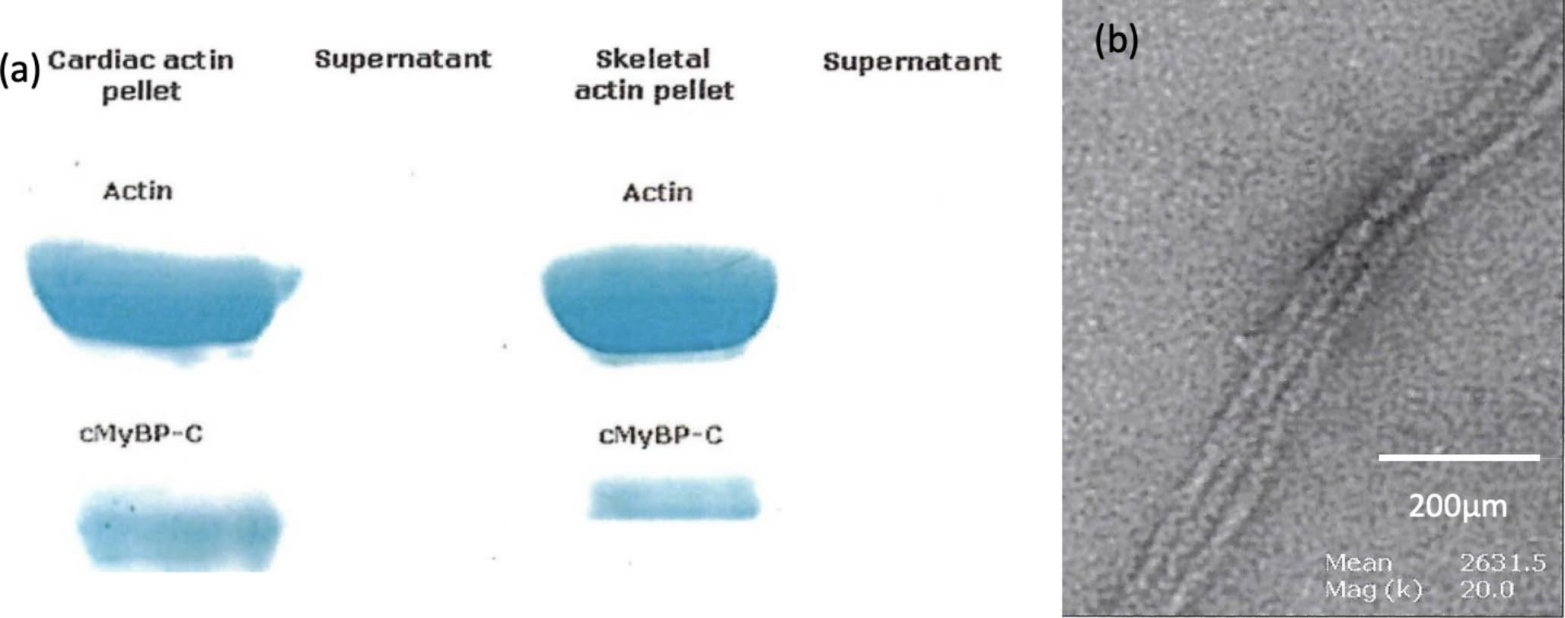
Co Sedimentation assays and Electron Microscopy of cMyBP-C on F-Actin (**a**) gel bands after spinning 1:1 F-actin with сМуВР-С. (**b**) Electron micrographs of a 1:1 F-actin with cMyBP-C

## Conclusions

Muscle proteins play an important part in vivo in a variety of cells through, for example, their structural/functional involvement in molecular motors, cellular function regulators and organisers (Hodgkinson 2000). Further, proteins in various muscle types have been shown to carry mutations associated with disease. It is therefore important to know the structures of the wild type proteins and, as a consequence, be able to determine the spatial locations of the various mutations and the stereochemical implications of the mutant sequences.

With the increasing technical advances in structural biology that have been made in recent years it is now possible to gain useful insights into the mechanism of action of many of the key proteins in muscle. Indeed, crystal structures of titin, tropomyosin, troponin, dystrophin and utrophin, to name but a few, have allowed a detailed understanding of their function to be gained. A key step, however, remains the ability of the protein to crystallise in a form suitable for X-ray diffraction analysis.

In this mini review we have shown while some proteins crystallise readily under many conditions, others appear to be recalcitrant to crystallisation regardless of the extent of the screening process employed. It is therefore possible for us to conclude that although empirical methods are often successful in producing crystals, various problems can arise that frustrate efforts in producing crystals suitable for X-ray analysis. These include the inability to reproduce a particular crystal form. To overcome the crystallisation bottleneck, one needs to go beyond the trial-and-error approaches. Towards that end, high quality diffracting crystals of cMyBP-C were produced when new and modified crystallisation methodologies were devised. Therefore, it remains a priority to continually develop new methods that enable the highest quality crystals to be obtained if the field of muscle research is to thrive in the future.
